# Deciphering how plant pathogenic bacteria disperse and meet: Molecular epidemiology of *Xanthomonas citri* pv. *citri* at microgeographic scales in a tropical area of Asiatic citrus canker endemicity

**DOI:** 10.1111/eva.12788

**Published:** 2019-04-10

**Authors:** Olivier Pruvost, Karine Boyer, Virginie Ravigné, Damien Richard, Christian Vernière

**Affiliations:** ^1^ CIRAD UMR PVBMT Saint Pierre, La Réunion France; ^2^ ANSES Saint Pierre, La Réunion France; ^3^ Université de la Réunion UMR PVBMT Saint Denis, La Réunion France; ^4^ CIRAD UMR BGPI Montpellier France

**Keywords:** genotyping, microsatellites, plant bacterial diseases, spatial structure

## Abstract

Although some plant pathogenic bacteria represent a significant threat to agriculture, the determinants of their ecological success and evolutionary potential are still poorly understood. Refining our understanding of bacterial strain circulation at small spatial scales and the biological significance and evolutionary consequences of co‐infections are key questions. The study of bacterial population biology can be challenging, because it requires high‐resolution markers that can be genotyped with a high throughput. Here, we overcame this difficulty for *Xanthomonas citri* pv. *citri*, a genetically monomorphic bacterium causing Asiatic citrus canker (ACC). Using a genotyping method that did not require cultivating the bacterium or purifying DNA, we deciphered the pathogen's spatial genetic structure at several microgeographic scales, down to single lesion, in a situation of ACC endemicity. In a grove where copper was recurrently applied for ACC management, copper‐susceptible and copper‐resistant *X. citri* pv. *citri* coexisted and the bacterial population structured as three genetic clusters, suggesting a polyclonal contamination. The range of spatial dependency, estimated for the two largest clusters, was four times greater for the cluster predominantly composed of copper‐resistant bacteria. Consistently, the evenness value calculated for this cluster was indicative of increased transmission. Linkage disequilibrium was high even at a tree scale, probably due to a combination of clonality and admixture. Approximately 1% of samples exhibited within‐lesion multilocus polymorphism, explained at least in part by polyclonal infections. Canker lesions, which are of major biological significance as an inoculum source, may also represent a preferred niche for horizontal gene transfer. This study points out the potential of genotyping data for estimating the range of spatial dependency of plant bacterial pathogens, an important parameter for guiding disease management strategies.

## 1 INTRODUCTION

Understanding the population biology of plant pathogens, that is, their population genetic structure and dynamics, is important for an informed management of plant diseases in agro‐ecosystems (Burdon & Thrall, 2008; Milgroom, 2015; Stukenbrock & McDonald, 2008). It has been proposed that the pathogens that would pose the greatest challenge for sustainable disease management were those with the greatest evolutionary potential, and thus those that exhibited high mutation rates, large effective population sizes, a high level of gene flow, and/or a mixed reproduction system (i.e., with an alternation of sexual and asexual phases; McDonald & Linde, 2002). However, there is growing concern that pathogens with a clonal or partially clonal mode of reproduction can cause major outbreaks on a regular basis and at potentially large spatial scales, especially in agro‐ecosystems (e.g., McDonald & Stukenbrock, 2016; Pennisi, 2010; Tibayrenc & Ayala, 2012). Many agro‐ecosystems are characterized by large‐scale uniformity of the environment, the host plant, and the agricultural practices. A direct consequence is that the main barrier to large‐scale outbreaks in intensive agro‐ecosystems is the initial adaptation step required for a pathogen population to be able to thrive in the biotic and abiotic conditions typical of a given cropping system. Adapting to a new crop or agricultural practice can be within the reach of species with a clonal or partially clonal mode of reproduction, particularly in situations where the new crop or cropping system is durably installed in an area of endemicity of the pathogen (Hufbauer et al., 2012; Stukenbrock & McDonald, 2008).

Once a pathogen population has adapted to a new crop or to new cropping conditions, the extent of the outbreak is likely determined by pathogen dispersal abilities in relation to landscape structure (i.e., the spatial distribution of agricultural practices and plant susceptibility) and weather conditions. Understanding how clonal and partially clonal pathogens initially adapt to new environmental conditions and, subsequently, propagate is key when it comes to anticipating large‐scale epidemics in agro‐ecosystems.

Many economically important plant diseases are caused by bacteria (Mansfield et al., 2012). Bacteria reproduce by binary fission, but a variety of mechanisms allows horizontal gene transfer (HGT) between bacteria that are more or less related (Popa & Dagan, 2011). Depending on the intensity of HGT and recombination, bacterial species can exhibit a population structure ranging from strictly clonal to panmictic (Maynard Smith, Smith, O' Rourke, & Spratt, 1993). Bacterial plant pathogens with a predominantly clonal mode of evolution are frequent, and some of them are even referred to as genetically monomorphic (Achtman, 2008). Monomorphic bacteria are those species for which no polymorphism can be detected on the housekeeping genes usually used for bacterial classification. Such species are considered as having a very low diversity on their core genome, and they are thus thought to rely on occasional HGT for rapid adaptation to new environmental constraints (Achtman, 2012).

Understanding how monomorphic bacteria adapt to new environmental conditions thus requires identifying how frequently and by which mechanisms the epidemiological dynamics allow genetically different bacteria to circulate and meet, potentially leading to HGT. To do so, it is crucial to gain an in‐depth knowledge of population structure at microgeographic scales, that is, from the field level down to the single lesion level (Linde, Zhan, & McDonald, 2002): (a) Landscape and field scales provide information on the pathogen's dispersal capacity; (b) the individual host level provides information on the pathogen's reproductive system; and (c) lesion scale provides information on the potential for competition and genetic interaction, including HGT among cells (Alizon, Roode, & Michalakis, 2013).

Genotyping has now gained some popularity for assessing patterns of dispersal in bacteria. The genetic structure of plant pathogens has been mostly studied at the level of the production basin (i.e., landscape genetics; Plantegenest, May, & Fabre, 2007; Rieux, Lenormand, Carlier, Bellaire, & Ravigné, 2013). Deciphering the genetic structure of bacterial populations at smaller spatial scales is challenging. The traditional genotyping of a number of housekeeping gene portions (referred to as multilocus sequence typing, MLST) lacks resolution, especially for monomorphic pathogens by definition (Achtman, 2008). In contrast, MultiLocus Variable number of tandem repeat Analysis (MLVA) has been shown to allow revealing infraspecific genetic diversity in several human monomorphic pathogens (Van Belkum, 2007; Lindstedt, 2005; Pourcel & Vergnaud, 2011). Recent developments in tandem repeat (TR) genotyping have included culture‐independent analyses of clinical samples, whereby direct MLVA typing from DNA extracted from clinical specimens improved the understanding of the genetic structure and transmission of several human bacterial pathogens, for example, *Bacteroides fragilis*, *Bordetella pertussis*, *Mycobacterium tuberculosis*, *Mycoplasma pneumoniae*, or *Mycoplasma hyopneumoniae* (Bidovec‐Stojkovic, Seme, Zolnir‐Dovc, & Supply, 2014; Bjerke et al., 2011; Litt, Jauneikaite, Tchipeva, Harrison, & Fry, 2012; Pereyre et al., 2012; Vranckx et al., 2011).

*Xanthomonas citri* pv. *citri* (synonym = *X. axonopodis *pv. *citri*) is the causal agent of Asiatic citrus canker (ACC), a striking example of a plant bacterial pathogen that poses a major economic risk to agriculture on several continents. It affects crop profitability, both directly (i.e., fruit yield and reduced quality) and indirectly (restrictions of fresh fruit exports because of quarantine regulations in countries not exposed to ACC; Graham, Gottwald, Cubero, & Achor, 2004). Generally, attempts to control the disease involve integrated management, which includes a combination of measures, such as establishing groves from disease‐free partially resistant nursery plants, using efficient windbreaks, avoiding overhead irrigation, spraying copper extensively when plant organs are highly susceptible, and pruning diseased shoots during grove maintenance operations (Gottwald, Graham, & Schubert, 2002).

*Xanthomonas citri* pv. *citri* exhibits very low diversity when sequencing a few housekeeping genes (Almeida et al., 2010; Bui Thi Ngoc et al., 2010) making it considered as a genetically monomorphic bacterial pathogen (Achtman, 2008). Its evolution is assumed to be predominantly clonal (i.e., recombination occurs in this organism but not on a sufficiently frequent basis to erase the clonal structure). This assumption is based on (a) a strong congruence between genetic distances among strains derived from totally unrelated genotyping techniques, which obviously explore different genome regions (Bui Thi Ngoc, Vernière, Jarne et al., 2009; Vernière et al., 2014), (b) the presence of multilocus associations in molecular epidemiology analyses, which are stable in space and time (Pruvost et al., 2014), and (c) genome‐wide analyses of recombination (Gordon et al., 2015; Zhang et al., 2015). The potential of *X. citri* pv. *citri* for rapid adaptation to environmental constraints (including pest management practices) has recently been demonstrated by the emergence of plasmid‐borne copper resistance. Copper resistance has been first identified in Argentina in the 1990s and two decades later in Reunion Island where the present study was conducted (Behlau, Canteros, Minsavage, Jones, & Graham, 2011; Richard et al., 2017).

*Xanthomonas citri* pv. *citri* infects citrus through stomata on juvenile organs and wounds (regardless of the organs' growth stage), which are associated with insect damage or grove maintenance operations (Graham et al., 2004). *Xanthomonas citri* pv. *citri* survives for long periods in canker lesions, mainly on leaves and branches, where populations may exceed 10^7^ cells per lesion (Gottwald et al., 2002). Apart from surface biofilms, which possibly represent a more minor inoculum source, the pathogen cannot survive outside canker lesions for extended periods (Cubero, Gell, Johnson, Redondo, & Graham, 2011; Graham et al., 2004). It is dispersed naturally within individual trees or between neighboring trees in droplets, by splashing or wind‐driven dispersal, resulting in aggregated disease patterns (Danos, Berger, & Stall, 1984). In addition to this regular short‐range propagation, extreme weather events and human activities sporadically cause long‐distance dispersal, which can have a major epidemiological impact (Irey, Gottwald, Graham, Riley, & Carlton, 2006).

The aim of the present study was to better understand how epidemiological dynamics determine the potential for HGT in a situation of ACC endemicity. To achieve this, we investigated the distribution of genetic variability at several microgeographic scales (i.e., grove, tree, branch, and single canker lesion), where bacterial cells interact, using a hierarchical sampling of ACC isolates in two citrus groves in Reunion Island. Recent molecular epidemiology studies on *X. citri* pv. *citri* revealed the high resolution of a new set of markers, MLVA‐14 (targeting 14 microsatellite loci) at local to regional scales (Leduc et al., 2015; Vernière et al., 2014). For a broader MLVA‐14 application, we describe a culture‐independent method for genotyping *X. citri* pv. *citri* directly from canker lesions. In this assay, DNA release and amplification are combined in a single step. It is performed directly from biological samples without the need for bacterial cultivation or genomic DNA extraction. By using this genotyping method, we addressed the following questions: How often do genetically distinct strains co‐occur at the field scale, at the tree scale, and in the same lesion? At the lesion scale, is this diversity strictly related to the population's clonal diversification (i.e., the observed diversity of descendants from the original haplotype, which initiated infection) and/or does it reveal the existence of polyclonal infections, which involve genetically distant strains? Consequently, what is the spatial genetic structure of *X. citri* pv. *citri* populations and what does it reveal about the heterogeneity of inoculum and the pathogen's transmission capability in citrus groves?

## 2 MATERIALS AND METHODS

### 2.1 Proof‐of‐concept experiment: bacterial strains and plant inoculation procedures

We used four pathotype A, genetic lineage 1 (DAPC 1) strains of *X. citri* pv. *citri *isolated from Kaffir lime (*Citrus hystrix*) in Réunion Island in 2010 (LH212, LH220, LH318, and LH339; Pruvost et al., 2014). Bacterial suspensions containing approximately 1 × 10^6^ CFU/ml were obtained from 10‐fold dilutions of spectrophotometrically adjusted suspensions (0.05 optical density OD_600_ ≈ 1 × 10^8^ CFU/ml) prepared in 0.01 M tris buffer pH 7.2 (Sigma 7–9, Sigma, Saint‐Quentin‐Fallavier, France) from 18‐hr‐old cultures on YPGA (yeast extract, 7 g/L; peptone, 7 g/L; glucose, 7 g/L; agar, 18 g/L; propiconazole, 20 mg/L; pH 7.2). Two strain mixes, (a) LH212/LH220 polymorphic at 10 TR loci and (b) LH318/LH339 polymorphic at four TR loci, were prepared from the suspensions with various relative concentrations (expressed as CFU/ml): strain 1 1 × 10^6^ / strain 2 1 × 10^6^, thereafter referred to as 1:1; 1 × 10^6^/1 × 10^5^, 10:1; 1 × 10^6^/1 × 10^4^, 100:1; 1 × 10^5^/1 × 10^6^, 1:10; 1 × 10^4^/1 × 10^6^, 1:100 (i.e., a total of 10 combinations). These mixes were infiltrated into the mesophyll of Kaffir lime leaves from the youngest vegetative flush (leaf size ranged from two‐thirds to three‐quarters of full expansion). For each strain combination, we inoculated eight sites (0.3–0.4 cm^2^) per leaf on duplicates, consisting of two leaves on different one‐year‐old potted plants. A suspension of each strain (i.e., unmixed) containing 1 × 10^6^ CFU/ml was inoculated as the genotyping control, as explained above. Leaves inoculated with tris buffer were used as the negative control. The experiment was replicated once. Kaffir lime plants were placed in growth chambers at 30 ± 1°C day and 26 ± 1°C night and 80 ± 5% relative humidity with a photoperiod of 12 hr for 22 days. Leaf disks centered on individual lesion were punctured with a 12‐mm sterile punch and individually soaked in 0.5 ml sterile tris buffer supplemented with 0.2% Tween‐20 in AB0661 microplates (Thermo Scientific, Illkirch, France) for 1 hr at room temperature under gentle (50 rpm) orbital shaking.

### 2.2 Direct MLVA‐14 genotyping

MLVA‐14 primers were used for multiplex PCR, as reported previously (Bui Thi Ngoc et al., 2009). These primers primarily produced amplicons at all or most loci for *X. citri* pathovars (Bui Thi Ngoc et al., 2009). In contrast, no amplification was produced from nonpathogenic *Xanthomonas* recovered from citrus assigned to species other than *X. citri* (data not shown; for a list of assayed isolates, see Delcourt et al., 2013 and Pruvost et al., 2015). Direct genotyping was achieved using the Clontech Terra PCR Direct Polymerase Mix (Ozyme, Saint‐Quentin‐en‐Yvelines, France). Briefly, 1 µl of biological sample (i.e., macerate obtained as described above) was used as the template in mixes containing 0.3 µM of each primer. Primers were labeled with one of the following fluorescent dyes: 6‐FAM; NED; PET; VIC (Applied Biosystems, Life Technologies, Courtaboeuf, France); 1× Terra PCR Direct Buffer (containing a non‐*Taq* DNA polymerase preblended with a monoclonal antibody that suppresses polymerase activity up to 98°C, allowing hot‐start PCR amplification from small amounts of template, dNTPs, and MgCl_2_); 0.5× Q‐solution (Qiagen, Courtaboeuf, France); and HPLC water to yield a final volume of 15 µl per reaction. PCR amplifications were performed in a GeneAmp PCR System 9700 Thermocycler (Applied Biosystems) under the following conditions: 2 min at 98°C for hot‐start activation; 35 cycles of denaturation at 98°C for 10 s, annealing at temperatures ranging from 64°C for primer pool 3 (XL3, XL5, XL9, XL14) and 4 (XL1, XL4, XL13, XL15) to 68°C for pool 1 (XL6, XL10, XL11) and 2 (XL2, XL7, XL8) for 15 s, extension at 68°C for 1 min; and a final extension step at 68°C for 30 min. Aliquots (1 µl) of amplicons diluted 1/40 to 1/100 were mixed to 10.9 µl of Hi‐Di formamide and 0.1 µl of a GeneScan 500LIZ internal lane size standard (Applied Biosystems). Capillary electrophoresis was performed in an ABI PRISM 3130xl genetic analyzer (Applied Biosystems) using performance‐optimized polymer POP‐7 at 15,000 V for ≈ 20 min at 60°C, with an initial injection of 21 s. Electropherograms were analyzed using Genemapper software 4.0 (Applied Biosystems). In the proof‐of‐concept experiments involving inoculum relative concentrations of 1:10, 10:1, 1:100, and 100:1, densitograms were standardized so that the height of the peak corresponding to the most concentrated strain was adjusted to 7,000 relative fluorescent units (rfu). Once standardization was achieved, peaks corresponding to the least concentrated strain were scored positive when their height exceeded 100 rfu. Strain IAPAR 306 of *X. citri* pv. *citri* was used as a positive control in each experiment (Da Silva et al., 2002).

### 2.3 cop PCR amplification

Fifty‐one and 27 *X. citri* pv. *citri* isolates collected in grove 1 and grove 2, respectively, were assayed for the presence of *copL* by PCR amplification from boiled bacterial suspensions. PCRs were mostly performed as reported previously (Behlau, Hong, Jones, & Graham, 2013). However, we used GoTaq Flexi Polymerase (Promega, Charbonnières‐les‐Bains, France), decreased the primer concentration (5 pmol/μl), and set the annealing temperature at 66°C. PCR amplification, using *copA* and *copB* primers, was also performed on a subset of samples with GoTaq Flexi Polymerase and the other conditions reported above. The copper‐resistant *X. citri* pv. *citri* strain LH201 was used as a positive control in each experiment (Richard et al., 2017).

### 2.4 Experimental groves

We obtained samples from two Kaffir lime orchards located in Petite‐Ile and Pierrefonds (Réunion Island), referred to as grove 1 and grove 2, respectively. The groves were 12 and 5 years old at the time of sampling. They were managed according to a standard integrated pest management scheme (windbreaks, copper sprays, and drip irrigation whenever necessary).

### 2.5 Diversity of *X. citri* pv. *citri* at various spatial scales

At least 75% of trees in each grove were randomly sampled for canker leaf lesions for assessing the diversity at grove scale. Up to five lesions per tree were assayed by direct MLVA‐14 genotyping (as described above), yielding a dataset in which MLVA‐14 data were obtained for 361 and 420 single canker lesions in groves 1 and 2, respectively. All symptoms were examined with a stereoscopic microscope to eliminate the ones that could represent two coalescing lesions. For each sample, unambiguous microsatellite data (i.e., when a single or two multilocus haplotypes differing at a single TR locus were identified) were used to assess the *X. citri *pv*. citri* population structure.

An intensive sampling of six trees in grove 2 (distant one to another by 13–50 m) was performed in order to assess the diversity at the branch and tree scales. Twenty‐nine to thirty three lesions from each of the three main branches of each analyzed tree were sampled, yielding a total number of samples per tree ranging from 91 to 96, which were subsequently analyzed as described above.

In order to assess polymorphism at the canker lesion scale, single‐colony isolation of *X. citri *pv*. citri* was performed on KC semi‐selective medium using 10‐fold dilutions of macerates from nine lesions (collected in grove 1), where polymorphism at >4 TR was recorded by direct MLVA‐14 genotyping (Pruvost, Roumagnac, Gaube, Chiroleu, & Gagnevin, 2005). Fifty‐eight to ninety colonies of *X. citri *pv*. citri* were recovered from each of these single canker lesions and were further assayed by MLVA‐14, as explained above.

Nei's index of gene diversity, clonal heterogeneity (Simpson index), and clonal evenness were calculated with the poppr 2.2.1 package in R (Kamvar, Tabima, & Grünwald, 2014). Allelic richness (*A*) and private allelic richness (*A*_p_) were computed using the rarefaction procedure for unequal sample sizes with HP‐RARE version 1.0 (Kalinowski, 2005).

Categorical minimum spanning trees were built using the algorithm recommended for TR data, combining global optimal eBURST (goEBURST) and Euclidean distances in PHYLOVIZ v1.1 (Francisco et al., 2012).

### 2.6 Population genetic structure

A hierarchical sampling was conducted in grove 2. Analyses at the branch scale were carried out on six selected trees. Using MLVA, we assessed the genetic structure of populations in grove 2, based on geographic locations from the branch to the orchard level. An analysis of molecular variance (AMOVA) was performed using the arlequin version 3.5 software package (Excoffier, Laval, & Schneider, 2005). The individuals were grouped according to their geographic origin (branch, tree or orchard). Levels of significance were determined by computing 999 random permutations.

We used different approaches to examine the genetic differentiation among populations (i.e., strains originating from a single site). Population pairwise *F*_ST_ and *R*_ST_ values were computed, and their significance was tested with 999 permutations using arlequin. *R*_ST_ may better represent differentiation when the mutational process follows a stepwise mutation model (Balloux & Lugon‐Moulin, 2002). A significant difference between *F*_ST_ and *R*_ST _estimates was tested, using the SPAGeDI version 1.5 software, based on a permutation procedure of allele sizes (1,000 randomizations) to determine whether stepwise‐like mutations contributed to genetic differentiation (Hardy & Vekemans, 2002). Comparing *F*_ST_ and *R*_ST _values can provide valuable insights into the main causes involved in population differentiation (Hardy, Charbonnel, Fréville, & Heuertz, 2003). *R*_ST_ is expected to be larger than *F*_ST_ when the effect of stepwise‐like mutations on differentiation is large as compared to that of drift and/or migration.

### 2.7 Spatial autocorrelation analysis

The spatial genetic structure (SGS) at the individual level (i.e., multilocus microsatellite genotypes [MLMGs]) was investigated using a multivariate approach to spatial autocorrelation analysis for multiallelic co‐dominant markers (Smouse & Peakall, 1999). Spatial autocorrelation analysis produces statistics describing how a variable (e.g., genetic distance) is autocorrelated through space. A measure of genetic relatedness, *F_ij_* or *r* (see below), is calculated between individuals for each possible pair that is a specified distance apart. These pairwise genetic coefficients are regressed on pairwise spatial distances. We computed mean values per distance interval. Spatial autocorrelation analyses were conducted on groves 1 and 2. Tree spacing was 4 m, both in and between rows. We defined different distance classes with a spatial lag ranging from 4 to 10 m. Analyses were performed using GenAIEx 6.5 (Peakall & Smouse, 2006) and SPAGeDI version 1.5 (Hardy & Vekemans, 2002). GenAIEx calculates an autocorrelation coefficient (*r*), which provides a measure of the genetic similarity between pairs of individuals belonging to the same distance class. An autocorrelogram plots *r* as a function of distance class. The assumption of absence of SGS was tested by random permutation of all individuals from each grove. We then estimated the 95% confidence of the null hypothesis of no spatial genetic structure around *r* after 999 permutations (Smouse & Peakall, 1999). SPAGeDI computes the Loiselle kinship coefficient (*F_ij_*). If *F*_(_ *_d_*_)_, the mean estimates of *F_ij_* over a given distance interval *d*, tends to decrease linearly with *d *or ln(*d*), the extent of SGS, referred to as the *Sp *statistic, can be quantified by the ratio −*b_F_/*(1 − *F*_(1)_), where *b_F_* is the regression slope of *F_ij_* on ln(*d_ij_*), separating the pairs of isolates, and *F*_(1)_ is the mean kinship coefficient of the first distance class (Vekemans & Hardy, 2004). The *Sp *statistic, which can be used to compare SGS among populations or species, is robust to the sampling scheme, at least as long as *F*(*d*) is approximately linearly proportional to *d *or ln(*d*) (Vekemans & Hardy, 2004). The 95% confidence interval of *F*(*d*) was estimated after 999 permutations. We tested the significance of the SGS by comparing the observed regression slope *b_F_* with those obtained under the null hypothesis, by permuting individual locations (*N* = 999). The standard error of the regression slope *b_F_* was obtained by jackknifing over loci (*N* = 999). It was used as an estimate of the variability of *Sp* (Hardy et al., 2006; Jump & Penuelas, 2007). We computed *Sp* for a distance class of 4 m (i.e., the distance between trees along and across rows) over the same range of 0–160 m (not enough pairs for comparisons over larger distances) using the whole datasets, and in the case of grove 2, independently for the two main genetic clusters identified (i.e., cluster 3 did not have enough pairs per distance class).

### 2.8 Linkage disequilibrium

Multilocus linkage disequilibrium (*r_d_*) was estimated for the MLVA data using the poppr package in R (Agapow & Burt, 2001; Kamvar et al., 2014). The significance of *r_d_* was tested by comparing the observed variance to the distribution of variance expected under the null hypothesis of panmixia (999 permutations). The nonrandom association of alleles was evaluated both on strains and on haplotypes (i.e., clone‐corrected datasets) to minimize the effect of frequent haplotypes in some local populations.

## 3 RESULTS

### 3.1 Proof‐of‐concept experiment

One of the prerequisites for the study was to test the ability of the new culture‐independent MLVA genotyping method to detect multiple infections. In this first experiment, we mimicked co‐infections of citrus leaves with bacterial strains, which exhibited polymorphism at the targeted loci (ranging from 1 to 14 TRs and amplicon size differences of 7–98 bp). Canker‐like lesions were produced by all strains and strain mixes, but not by the negative controls. All the samples with equi‐concentrated bacterial suspensions produced the expected amplicons. When differences in TR numbers among strains were ≤2 at a given locus, the peak height ratio of amplicons was around 1. This suggests that the PCRs were equally effective, irrespective of the allele. As expected for the TR differences >2, the PCR amplification was more efficient for the shortest allele (Table 1). Similarly, when a 10‐fold difference in bacterial suspension concentrations was used in the mixes, the expected amplicons were observed for all samples. This was the case even when the strain with the largest number of the considered TR was adjusted to the lowest bacterial concentration. In contrast, this result was not obtained when there was a 100‐fold difference in bacterial suspension concentrations (data not shown). Under these conditions, the peaks with the expected lowest height were only detected sporadically.

**Table 1:** Peak height ratio of polymorphic tandem repeat amplicons obtained by direct MLVA‐14 genotyping and derived from co‐inoculations of Kaffir lime leaves with pairs of *Xanthomonas citri* pv. *citri* haplotypes

TR locus^a^	Strain mix	Size difference^b^	Ratio^c^	
			Experiment #1	Experiment #2
XL1	LH212/LH220	98 (14)	8.99 (3.50)	11.33 (2.53)
XL2	LH212/LH220	7 (1)	1.16 (0.22)	1.01 (0.14)
XL3	LH212/LH220	7 (1)	1.05 (0.25)	0.90 (0.16)
XL3	LH318/LH339	14 (2)	1.13 (0.23)	1.17 (0.24)
XL4	LH212/LH220	14 (2)	1.18 (0.29)	0.95 (0.08)
XL5	LH318/LH339	7 (1)	1.27 (0.25)	0.99 (0.02)
XL6	LH212/LH220	70 (10)	2.68 (0.67)	2.40 (0.62)
XL6	LH318/LH339	7 (1)	1.02 (0.07)	1.03 (0.13)
XL7	LH212/LH220	14 (2)	1.36 (0.73)	0.98 (0.15)
XL8	LH212/LH220	7 (1)	1.16 (0.23)	1.39 (0.32)
XL10	LH318/LH339	14 (2)	1.04 (0.12)	1.00 (0.08)
XL11	LH212/LH220	7 (1)	1.02 (0.13)	0.99 (0.01)
XL14	LH212/LH220	12 (2)	1.28 (0.31)	1.14 (0.15)
XL15	LH212/LH220	21 (3)	1.56 (0.39)	1.28 (0.31)

a According to Bui Thi Ngoc et al. (2009).

b Amplicon size difference in bp and corresponding number of tandem repeat difference (in brackets).

c Mean ratio (*n* = 12) and standard deviation (in brackets).

### 3.2 Population structure at a grove scale

The two groves under study were established with Kaffir lime plants (*C. hystrix*), some of which were contaminated in the nursery prior to grove establishment. Disease incidence in both orchards was high (>0.8), in agreement with the species' high susceptibility to *X. citri* pv. *citri* pathotype A (Gottwald et al., 2002). At the time of sampling, disease severity was approximately ten times higher in grove 2 than in grove 1. Differentiation was highly significant between the *X. citri* pv. *citri* populations from the two groves (*R*_ST_ = 0.697, *p* < 0.001 and *F*_ST_ = 0.274, *p* < 0.001). At this scale, permutation test analyses in SPAGeDI indicated that *R*_ST_ was significantly different from *F*_ST_ (*p* < 0.001). This suggests that allele size was informative for assessing population differentiation.

In grove 1, we analyzed 369 MLMGs, which separated as 357 distinct haplotypes, corresponding to a mean allelic richness of 8.72 (private allelic richness 2.79). Evenness was 0.961, indicating that the frequencies of the different haplotypes were quite similar. Haplotypes structured as 44 clonal complexes (CC), that is, networks of single‐locus variants (SLV), and 213 singletons, that is, haplotypes with no identified SLV, the latter representing 59% of the samples. The largest CC in size consisted of 31 haplotypes (9% of the samples analyzed). In grove 2, the structure was markedly different. The 409 MLMGs analyzed were separated as 273 distinct haplotypes, corresponding to a mean allelic richness of 7.28 (private allelic richness 1.35). They structured as 22 CCs and 53 singletons, the latter representing only 13% of the samples. The largest CC in size consisted of 105 haplotypes, including 54% of the samples analyzed. Some haplotypes were strongly overrepresented in the dataset, which yielded an evenness of 0.417.

Genetic clusters were delineated from the produced minimum spanning trees. The clusters were defined so that each cluster contained haplotype networks linking up to quadruple‐locus variants. When these cluster delineation rules were applied, data from grove 1 yielded a single cluster and eight singletons, each composed of a single sample (Supporting Information Figure S1). In contrast, three clusters were identified in grove 2, namely cluster 1 (number of samples *n*_1_ = 306), cluster 2 (*n*_2_ = 62), and cluster 3 (*n*_3_ = 41) (Supporting Information Figure S2). Pairs of haplotypes bridging clusters differed at five microsatellite loci by one to 11 repeats. Although allelic richness was similar for each cluster detected in grove 2 (3.21 ≤ *A* ≤ 3.81), evenness was markedly different for cluster 1 (0.410), as compared to clusters 2 (0.944) and 3 (0.923). This suggests that the heterogeneous haplotype frequencies identified in grove 2 were actually restricted to cluster 1.

Assays for the presence of *copL*, based on PCR amplification from *X. citri* pv. *citri* cultures (subsequently collected in both groves), indicated the widespread presence of copper resistance in grove 2 (19/27; ≈70%). Copper resistance was not detected in grove 1 (*n* = 51). All strains collected from grove 2 were subject to microsatellite genotyping. Interestingly, all copper‐resistant strains grouped in cluster 1, while copper‐susceptible strains split into clusters 1 (*n* = 2), 2 (*n* = 4), and 3 (*n* = 2).

### 3.3 Spatial genetic structure

**Figure 1:**
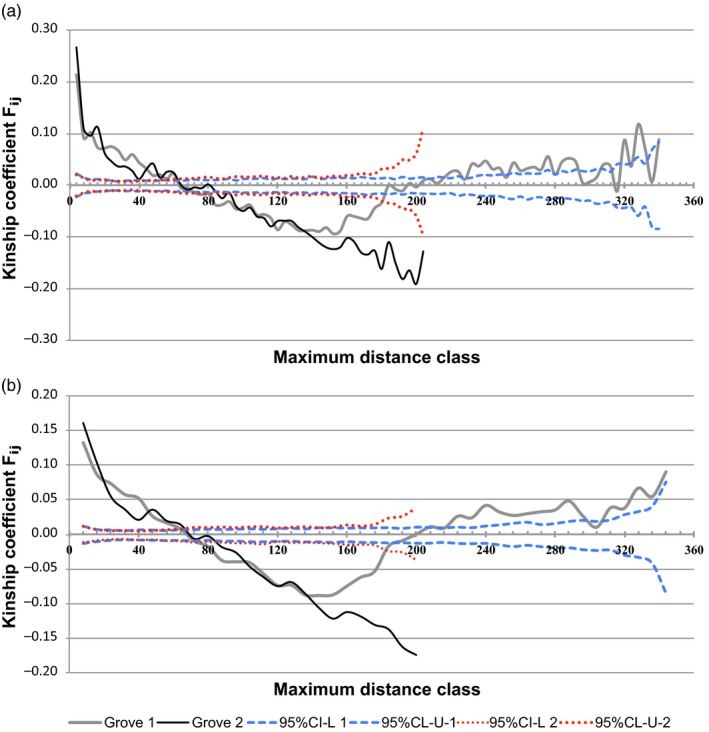
Spatial autocorrelograms plotting the mean kinship coefficients *F_ij_* per distance class (solid lines) and 95% confidence intervals (upper U and lower L bounds in dashed lines). The estimations were calculated after 999 permutations about the null hypothesis of a random distribution of *Xanthomonas citri* pv. *citri* in two Kaffir lime groves. Results correspond to distance class sizes of 4 m (a) and 8 m (b). Individuals are significantly more similar than would be expected by random distribution, when *F*(*d*) is above the 95% confidence limit

The spatial genetic structure of MLMGs within groves 1 and 2 was calculated using SPAGeDI. It revealed that the kinship coefficients (*F_ij_*) decreased continuously with increasing spatial distances between pairs of samples for groves 1 and 2 (Figure 1). In both groves, the highest positive *F_ij_* was recorded for the first distance class. *F_ij_* was positive and significantly larger than expected for a random spatial distribution for maximal distances up to approximately 60–70 m (estimated with distance classes ranging from 4 to 10 m; Table 2). *F_ij_* was negative and statistically significant, indicating that MLMGs were genetically independent over distances of 72–180 m and 96–204 m in groves 1 and 2, respectively. The regression slopes differed significantly from the results expected for the hypothesis of a random spatial distribution of isolates (*p* < 0.001) (Table 2). Analyses using the autocorrelation coefficient *r* in GenAIEx produced very similar results (Table 2, Supporting Information Figure S3).

**Table 2:** Estimates of spatial autocorrelation in grove 1 (range 0–348 m) and grove 2 (range 0–208 m), based on MLMGs identified using 14 microsatellite loci

Grove	Class size (m)	Pairs^a^	SPAGeDI						GenAIEx		
			Loiselle kinship coefficient *F_ij_* b		b‐log^c^	*Sp* d	Distance class for nonsignificant *F_ij_* e (m)	*p* f	Autocorrelation coefficient^b^		Distance class for observing nonsignificant *r* e (m)
			*F*_(1)_	*F*_(2)_					*r*_(1)_	*r*_(2)_	
1		67,896									
	4		0.215	0.091	−0.032	0.089 ± 0.020	56–60	<10^−3^	0.274	0.143	60–64
	6		0.167	0.096	−0.032	ND	66–72	<10^−3^	0.213	0.129	66–72
	8		0.133	0.086	−0.032	ND	72–80	<10^−3^	0.188	0.117	64–72
	10		0.122	0.077	−0.032	ND	70–80	<10^−3^	0.159	0.113	70–80
2		83,436									
	4		0.266	0.109	−0.076	0.098 ± 0.009	60–64	<10^−3^	0.429	0.255	68–72
	6		0.210	0.099	−0.076	ND	66–72	<10^−3^	0.359	0.231	72–78
	8		0.160	0.103	−0.076	ND	72–80	<10^−3^	0.314	0.238	72–80
	10		0.148	0.084	−0.076	ND	70–80	<10^−3^	0.305	0.190	70–80
Cluster 1	4	46,665	0.280	0.076	−0.071	0.101 ± 0.007	36–40	<10^−3^	ND	ND	ND
Cluster 2	4	1891	0.250	0.193	−0.046	0.064 ± 0.011	8–12	<10^−3^	ND	ND	ND

Two genetic clusters from grove 2 were further analyzed separately.

ND, not done.

a Number of pairwise comparisons.

b *F_ij_*: Loiselle kinship coefficient; *F*_(1)_: mean kinship coefficient for the first distance class, that is, from all pairs of individuals that were 0–4 m apart for a class size of 4 m, *F*_(2)_: mean kinship coefficient for the second distance class (i.e., all the pairs of individuals that were >4 to 8 m apart for a class size of 4 m); *r*: autocorrelation coefficient for the first (1) and second (2) distance classes.

c Regression slope of pairwise kinship values on the logarithm of spatial distances.

d *Sp* statistics estimated for the distance class of 4 m over the same range of 0–160 m.

e Shortest distance class in which *F*(*d*) (i.e., the mean estimates of *F_ij_* over a given distance interval d, as determined by SPAGeDI) or *r* (i.e., the autocorrelation coefficient that provides a measure of the genetic similarity between pairs of individuals belonging to the same distance class, as measured by GenAIEx) is observed to be not significantly different from 0 (=no spatial genetic structure).

f Probability for a two‐tailed test for positive autocorrelation.

The values for the *Sp* statistic, which reflects the decreasing rate of pairwise kinships over distance, are less sensitive to the sampling scheme (Vekemans & Hardy, 2004). They were similar for groves 1 and 2 with *Sp* = 0.089 ± 0.020 and 0.098 ± 0.009, respectively (Table 2).

The spatial autocorrelation analysis for two of the three genetic clusters identified in grove 2 produced similar highly positive kinship coefficients for the first distance class. These decreased steadily over distance (Table 2). Significant positive *F_ij_* values were observed for clusters 1 and 2 over distance classes of up to 36–40 m versus 8–12 m, respectively. The *Sp* statistics were significantly different over a distance range of 0–160 m (0.101 ± 0.007 vs. 0.064 ± 0.011 for clusters 1 and 2, respectively). This suggests that cluster 1 has a stronger spatial genetic structure.

### 3.4 Population structure at a tree scale

Allelic richness ranged from 2.50 to 4.00 and from 1.57 to 2.28 at an individual tree and secondary branch scale, respectively (Table 3). Thirty to 36 haplotypes were distinguished for each tree with more than 80% of them being genetically tightly related, that is, a majority of SLVs and a few double‐locus variants (DLVs). In one case only (PF315), all the haplotypes belonged to a single clonal complex. The allele size permutation tests revealed that *R*_ST_ was not significantly different from *F*_ST_ at an individual tree (0.325 vs. 0.330, *p* = 0.610) or secondary branch scale (0.359 vs. 0.381, *p* = 0.569). All pairwise *R*_ST_ values observed among *X. citri* pv. *citri* populations sampled at a tree scale were significant (*p* < 0.05) (Supporting Information Table S1). The rate of pairwise *R*_ST_ comparisons between branch populations originating from distinct trees that were strongly differentiated (*p* < 0.01) was 90%, but dropped to 44% for branch populations sampled from the same tree (Supporting Information Table S2). The hierarchical analysis of molecular variance (AMOVA) showed that most of the genetic variance (62%) occurred within branches. Variation within individual trees and between trees represented an additional 8% and 30% of the total variance, respectively (Supporting Information Table S3).

**Table 3:** Diversity indices and multilocus linkage disequilibrium estimated by *r_d_* at tree and branch scales for six trees in grove 2, based on MLVA‐14 data

Scale level	*N* a	*N*_H_	*H*_E_	*A*	*A*_p_	Lambda	Evenness	Full datasets		Clone‐corrected datasets	
								*r_d_*	*p* value	*r_d_*	*p* value
Tree 1 (PF240)	93	36	0.353	4.00	0.36	0.350	0.605	0.351	<0.001	0.214	<0.001
Branch 1	30	13	0.280	2.79	0.07	0.270	0.590	0.539	<0.001	0.399	<0.001
Branch 2	31	13	0.399	2.57	0.21	0.386	0.834	0.557	<0.001	0.420	<0.001
Branch 3	32	17	0.308	3.28	0.07	0.299	0.568	0.194	<0.001	0.155	<0.001
Tree 2 (PF212)	93	35	0.275	3.21	0.21	0.272	0.586	0.091	<0.001	0.044	0.003
Branch 1	32	14	0.196	2.21	0.07	0.189	0.609	0.110	<0.001	0.067	0.021
Branch 2	30	13	0.262	2.29	0.07	0.253	0.661	0.171	<0.001	0.140	<0.001
Branch 3	31	15	0.244	2.28	0.07	0.236	0.625	0.168	<0.001	0.042	0.030
Tree 3 (PF251)	91	31	0.430	3.36	0.36	0.420	0.810	0.385	<0.001	0.309	<0.001
Branch 1	31	15	0.370	2.57	0.14	0.360	0.730	0.345	<0.001	0.271	<0.001
Branch 2	30	15	0.398	2.57	0.07	0.385	0.821	0.528	<0.001	0.418	<0.001
Branch 3	30	9	0.372	2.43	0.07	0.360	0.801	0.552	<0.001	ND	
Tree 4 (PF274)	91	31	0.229	2.57	0.00	0.227	0.609	0.165	<0.001	0.082	<0.001
Branch 1	31	12	0.101	1.86	0.00	0.098	0.516	−0.061	0.996	−0.101	0.996
Branch 2	29	9	0.183	1.86	0.00	0.176	0.633	0.244	<0.001	ND	
Branch 3	31	16	0.270	2.21	0.00	0.260	0.710	0.162	<0.001	0.101	0.002
Tree 5 (PF 307)	96	32	0.178	3.50	0.29	0.176	0.462	0.435	<0.001	0.362	<0.001
Branch 1	33	15	0.157	2.42	0.07	0.152	0.499	0.383	<0.001	0.363	<0.001
Branch 2	32	13	0.234	2.50	0.07	0.226	0.584	0.715	<0.001	0.617	<0.001
Branch 3	31	15	0.139	2.71	0.14	0.135	0.453	0.282	<0.001	0.238	<0.001
Tree 6 (PF315)	93	30	0.135	2.50	0.14	0.133	0.488	0.017	0.098	−0.052	1.000
Branch 1	30	10	0.094	1.57	0.00	0.091	0.570	−0.007	0.539	ND	
Branch 2	30	11	0.072	1.64	0.00	0.070	0.505	−0.054	0.899	ND	
Branch 3	33	15	0.201	1.92	0.14	0.195	0.674	0.043	0.014	−0.031	0.790

a *A*: allelic richness; *A*_p_: private allelic richness;* H*_E_: Nei's gene diversity; lambda: Simpson's index;* N*: number of samples; *N*_H_: number of haplotypes.

ND: not done when *N*_H_ < 12.

### 3.5 Linkage disequilibrium at a tree scale

Based on the multilocus *r_d_* index, the null hypothesis of linkage equilibrium (*r_d_* = 0) was rejected for populations from grove 1 (*r_d_* = 0.040, *p* < 0.001) and grove 2 (*r_d_* = 0.171, *p* < 0.001). It was also highly significant when estimated using clone‐corrected datasets (*r_d_* = 0.033, *p* < 0.001 and *r_d_* = 0.113, *p* < 0.001 for groves 1 and 2, respectively). Using both types of datasets for populations sampled from individual trees, except PF315 (Table 3), the *r_d_* was significantly different from zero at the tree level in grove 2. At the second‐order branch level, 14 out of 18 branch populations displayed *r_d_* ≠ 0 (Table 3). In a small number of datasets, *r_d_* was not different from zero. This corresponded to cases of very low genetic diversity, as revealed by diversity indices (Table 3) and minimum spanning trees (not shown). Datasets from which significant linkage disequilibrium was revealed consisted of two to ten entities (CC or singleton) with haplotypes each separated by up to decuple‐locus variations and often assigned to distinct clusters (grove 2). Therefore, even at a small spatial scale, such as a tree or secondary branch, admixture occurs frequently and is likely to interfere with the estimate of linkage disequilibrium.

### 3.6 Diversity of *X. citri* pv. *citri* in single canker lesions

**Figure 2:**
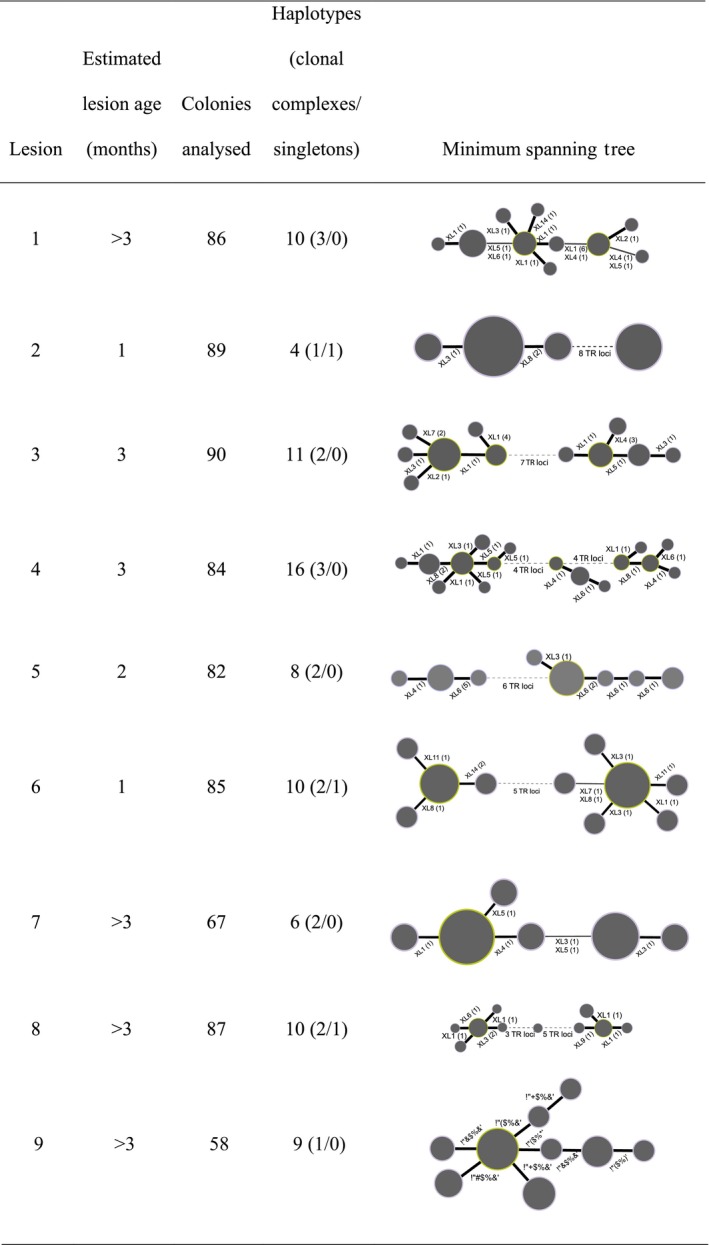
Minimum spanning trees derived from MLVA‐14 data, obtained from bacterial colonies recovered from nine single canker leaf lesions. Direct genotyping indicated some polymorphism at >4 tandem repeat loci. Dots represent haplotypes, and their diameter is representative of the number of strains per haplotype. Thick and thin plain lines between haplotypes indicate single‐ and double‐locus variations, respectively. Text along the links indicates which microsatellite(s) distinguished haplotypes. Figures in brackets indicate the number of repeats distinguishing haplotypes. Dashed lines link haplotypes differing at more than two microsatellite loci, and the number of polymorphic loci is shown along

All samples consisting of macerates from single canker lesions (*N* = 781) were successfully genotyped at all TR loci. Most MLMGs from assayed lesions appeared monomorphic (286 out of 361, i.e., 79%, and 335 out of 420, i.e., 80%, in groves 1 and 2, respectively). The observed polymorphism most often consisted of single‐locus (41 out of 75, i.e., 55%, and 37 out of 85, i.e., 44%, in groves 1 and 2, respectively) and single‐repeat (30 out of 41, i.e., 70%, and 24 out of 37, i.e., 65%, in groves 1 and 2, respectively) variations. However, multiple‐locus variations were also observed within single canker lesions (Supporting Information Figure S4). Single‐colony isolations were performed from single canker lesion macerates (collected in grove 1) for which direct genotyping suggested polymorphism at >4 TR loci. The populations obtained (*n* = 9, totaling 728 bacterial strains recovered from nine lesions) were subject to MLVA‐14. The bacterial population was structured as a CC only in a single lesion (#9 in Figure 2). Populations from two additional lesions (#1 and 7 in Figure 2) were composed of several CCs. The link between CCs consisted of double‐locus variations. In this type of situation, a single evolutionary step was missing. Therefore, we cannot exclude the possibility of incomplete sampling. For cases like this, it was unclear whether the observed polymorphism corresponded to diversification over time or to co‐infections of closely related haplotypes. However, for all other lesions, the minimum spanning tree suggested the possibility of co‐infection, due to the detection of distantly related haplotypes (Figure 2).

## 4 DISCUSSION

Here, we investigated the epidemiological dynamics of *X. citri* pv. *citri* at microgeographic scales using a molecular epidemiology approach combining microsatellite genotyping and an extensive hierarchical sampling of two citrus groves.

### 4.1 Culture‐independent microsatellite genotyping is useful for high‐throughput population analyses of Xanthomonas citri pv. citri

In previous studies, we demonstrated the potential of microsatellites for the analysis of the population structure and molecular epidemiology of *X. citri* pv. *citri* at local to regional scales (Leduc et al., 2015; Pruvost et al., 2015; Vernière et al., 2014). In the present study, using a simple and efficient culture‐independent method allowing to genotype the pathogen directly from canker lesions, we showed that this genotyping technique has a discriminatory power high enough for investigating microgeographic scales and detecting population diversification within lesions or polyclonal infections. The main limitation is the possibility that strains, which would have a very strong fitness difference (that reaches the two log limitation of our genotyping technique), could cohabit in a canker lesion. This coexistence of strains with much contrasted fitnesses is not expected in most epidemiological situations. The most comprehensive study on fitness variability among *X. citri* pv. *citri* strains was reported from a study on pummelo cultivars (*Citrus maxima*) in Japan. The population size of strains differing in aggressiveness due to the presence/absence of a plasmid encoding for an important pathogenicity effector was measured *in planta*, and the differences between strains were consistently found lower than the two log limitation of our technique (although no co‐inoculations of both strain types were tested; Shiotani, Fujikawa, Ishihara, Tsuyumu, & Ozaki, 2007).

### 4.2 Fine‐scale genetic structure corroborates direct dispersal observations

Estimating the dispersal ability of plant pathogenic bacteria is extremely important, as well as challenging for disease management. It can be estimated using spatio‐temporal analyses of disease incidence or severity data. However, this usually requires specific situations, for example, epidemic fronts, where it can be safely assumed that the inoculum is a rather genetically uniform entity. For instance, pioneering analyses of disease severity indices conducted in Argentina not long after the emergence of *X. citri* pv. *citri* showed that lesions were highly spatially aggregated. This strategy made it possible to monitor disease development in time and space “without the interpretative problems associated with an endemic disease” (Danos et al., 1984). In contrast, in endemic areas and, more generally, in outbreak areas where genetically distinct pathogenic populations coexist, the genetic relationships between lesions must be traced to estimate the dispersal distances (Leduc et al., 2015; Vernière et al., 2014), which requires sufficiently variable markers and an important sampling effort.

Our results clearly indicated an isolation‐by‐distance (IBD) pattern at a small scale within groves. In both groves, the highest positive *F_ij_* were recorded for the first distance class. Under conditions not involving extreme weather events, the marked decrease in kinship coefficients over geographic distance confirmed a limited dispersal ability of the pathogen, consistent with aggregated disease patterns previously reported for Asiatic canker (Gottwald et al., 2002). Interestingly, the analyses conducted at a tree and branch scale emphasized that most of *X. citri* pv. *citri* diversity, resulting from several evolutionary forces including mutation and migration (i.e., multiple introductions of the pathogen possibly occurred in the grove under study), was observed at an infrabranch scale.

### 4.3 A more efficient transmission of copper‐resistant strains

Microsatellite data collected from grove 2 revealed population heterogeneity and differences in terms of transmission abilities. This finding was probably related to the heterogeneous origin of the nursery citrus plants (used for grove establishment) and the presence of several ACC‐infected sites in the vicinity of grove 2. Copper resistance, first reported in Reunion Island in 2014 (Richard et al., 2017), was detected in grove 2, but not in grove 1. Our data suggested that copper‐resistant strains were restricted to a single cluster (cluster 1), in which they constituted a large majority. Interestingly, this cluster showed a very low evenness. It was primarily structured as a large CC and, therefore, highlighted clonal expansion. The characteristics of the two other clusters detected in grove 2 were similar to those described for grove 1. This suggested that active transmission was primarily a characteristic of copper‐resistant strains under the prevailing environmental conditions (i.e., no weather event likely to cause medium‐ to long‐distance spread and regular copper applications). This was further supported by the spatial autocorrelation data, which showed that the extent of spatial dependency (the distance for which lags were significantly autocorrelated) was four times larger for cluster 1 than for cluster 2. Secondary infections by *X. citri *pv*. citri* can occur if free water is available on plant surfaces. The presence of copper ions in free water on trees largely restricts secondary infections by copper‐susceptible strains. The present study is an example of anthropogenic activities driving changes in the population structure of a pathogen in an agro‐ecosystem (Thrall et al., 2011).

### 4.4 Canker lesions host polyclonal populations

Herein, the hierarchical analysis of molecular variance suggested that most of the genetic variation was found at the infrabranch scale, possibly making genetically variable *X. citri *pv. *citri* cells available for co‐infections of single stomata. It is not yet clear how common it is for genetically distinct strains of the pathogen to live in sympatry on the same plant organ or even in the same canker lesion. A very recent study indicated the presence in the same leaf sample of two strains with a chromosome differing by 52 SNPs. Signs of rearrangements and recombination of some plasmid‐borne TALE genes were also detected in these two strains (Gochez et al., 2018). Here in both studied groves, it was found that approximately 80% of the assayed lesions were monomorphic for the 14 microsatellite loci considered. Nearly half of the observed polymorphism consisted of SLVs (most of which consisting of single‐repeat variations), suggesting monoclonal infection followed by clonal diversification. Co‐infections by SLVs might have occurred given that SLVs can be available concomitantly for the infection process. Multiple infections at the scale of single lesions by genetically distinct strains occurred at a frequency of ca. 1%. This suggests that multiple infections likely occur many times in the course of an outbreak. Polyclonal infections could be the consequence of the availability of genetically diverse strains (at small scales) in a context of disease endemicity. Upon infection, genes encoding exopolysaccharides are strongly overexpressed. This yields bacterial cells that are embedded in a dense polysaccharide matrix in canker lesions and are available for secondary infections (Jalan et al., 2013). Although it has been shown experimentally that a single *X. citri* pv. *citri* cell can induce a canker lesion when introduced into a stoma (Gottwald & Graham, 1992), it is likely that exopolysaccharide‐embedded *X. citri* pv. *citri* micro‐aggregates, consisting of multiple cells, can penetrate plant openings and cause infection.

Overall, *X. citri* pv. *citri* displays a low ability to survive asymptomatically outside its hosts (Graham et al., 2004). Asymptomatic survival of *X. citri* pv. *citri* on citrus surfaces probably occurs at small population sizes, preferentially in bacterial biofilms (Cubero et al., 2011; Redondo et al., 2015). Canker lesions undoubtedly represent a preferred niche for *X. citri* pv. *citri* survival. They probably provide favorable conditions for HGT, that is, high bacterial concentrations of both donor and recipient strains in biofilm‐like structures within colonized tissues (Rigano et al., 2007).

### 4.5 Multiple infections may be key for adaptation of genetically monomorphic bacteria

Multiple infections by distinct pathogens can modify the extent of damage caused to crops in a positive (e.g., synergism between plant pathogenic microbes) or negative way and are often associated with changes in pathogen load. Gaining an improved knowledge of the impact of such multiple infections is a promising area for future (Tollenaere, Susi, & Laine, 2016). Multiple infections can have several consequences on bacterial populations. They would favor the selection of fitter haplotypes as a result of competition for plant resources and also promote HGT and genetic rearrangements, which are drivers of adaptation (Bartoli, Roux, & Lamichhane, 2016). In *X. citri* pv. *citri*, some genes linked to pathogenicity or growth ability were found on conjugative or mobilizable plasmids (Li & Wang, 2011; Yan & Wang, 2012). In situations (e.g., India, South‐East Asia) where distinct citrus‐pathogenic *X. citri* pv. *citri* pathotypes (i.e., infrapathovar variants that differ in terms or host range or pathological reaction type) coexist, lesions may also be privileged sites for DNA exchange between genetically distant strains, which share the same niche. Plasmids encoding the major TALE pathogenicity gene *pthA* were transferable between *X. citri* pv. *citri* and *Xanthomonas citri* pv. *aurantifolii*, two citrus pathogens (El Yacoubi, Brunings, Yuan, Shankar, & Gabriel, 2007). Similarly, several genomic regions, thought to have undergone recombination in *X. citri* pv. *citri* pathotype A^w^, share a very high genetic relatedness with corresponding regions in *Xanthomonas citri* pv. *bilvae. *This bacterium is also pathogenic to rutaceous species, but has a distinct symptomatology. Moreover, apart from potential pseudogenes, bacteriophages, and insertion sequences, the differences detected in terms of the gene content between *X. citri* pv. *citri* pathotypes corresponded to gene islands and regions of recombination, several of which are likely to have a plasmid origin (Gordon et al., 2015). Emergence of copper‐resistant *X. citri* pv. *citri* populations through HGT of a large conjugative plasmid is another striking example of such an acquisition of adaptive traits in response to the selective pressure by copper sprays widely used for controlling plant bacterial diseases (Richard et al., 2017).

Few studies on plant‐associated soilborne or epiphytic bacteria investigated polyclonal inocula within a same niche and genetic exchanges (Bailly, Olivieri, Mita, Cleyet‐Marel, & Béna, 2006; Bjorklof, Suoniemi, Haahtela, & Romantschuk, 1995). In contrast, pathological and evolutionary consequences of multiple infections by different strains are well known in virus evolution, be they animal‐ or plant‐associated viruses (Martin, Biagini et al., 2011; Martin, Lefeuvre et al., 2011). Similarly, several studies emphasized the presence of infraspecific fungal genetic diversity within a single lesion (Linde et al., 2002; McDonald, Zhan, & Burdon, 1999; Perez, Slippers, Wingfield, Hunter, & Wingfield, 2010). Among bacteria, the pathological and evolutionary consequences of multiple infections by members of a single species have been more extensively studied for human or animal pathogens, although the research efforts on such questions appear largely taxon‐dependent. A large number of studies involved mycobacterial species and especially two of them, *Mycobacterium tuberculosis* and *M. bovis *(Cohen et al., 2016; Ghielmetti et al., 2017; Navarro et al., 2015). In the case of human tuberculosis, the significance of multiple infections clearly is a growing concern especially in areas of high disease prevalence, with recent research aiming at accurately evidencing them, and estimating their consequences at both an individual level and population level on key factors, such as the maintenance and transfer of antibiotic resistance and the success of vaccination strategies (Bryant et al., 2013; Cohen et al., 2012; Mathema, Kurepina, Bifani, & Kreiswirth, 2006).

The importance of multiple infections in pathosystems involving plant pathogenic bacteria remains mostly nondocumented. Improving our knowledge on their biological significance and consequences on bacterial evolution will be an interesting challenge for the incoming years. In terms of bacterial plant disease control, multiple infections appear an important feature to account for. All management options (typically implemented as integrated control programs) that are likely to minimize gene or genotype flow and multiple infections (e.g., improved sanitation through the establishment of crop fields from pathogen‐free plant propagative material and physical elimination of inoculum, cultural practices minimizing pathogen transmission such as increased host heterogeneity, drip irrigation systems, efficient windbreak networks) should be promoted to improve the durability of control. New management strategies creating an increased crop plant and environmental heterogeneity of agro‐ecosystems are desirable options for the future of agriculture (McDonald & Stukenbrock, 2016).

## CONFLICT OF INTEREST

None declared.

## Data Availability

Microsatellite genotyping datasets are available in the CIRAD Dataverse (https://dataverse.cirad.fr/dataverse/pvbmt) as https://doi.org/10.18167/DVN1/NB0QMX.
