# Supplementary material for: Deciphering how plant pathogenic bacteria disperse and meet: Molecular epidemiology of Xanthomonas citri pv. citri at microgeographic scales in a tropical area of Asiatic citrus canker endemicity
Source: Evol Appl. 2019 Apr 10;12(8):1523–38. doi: 10.1111/eva.12788 (PMC6708428; doi:10.1111/eva.12788)

Fig. S2. Categorical minimum spanning tree, representing grove 2 MLMGs (n = 409; 273 haplotypes), based on microsatellite data. Dot diameter is representative of the number of samples per haplotype. Polymorphisms were represented as follows: single-locus variations = thick solid lines; double-locus variations = thin solid lines; triple-locus variations = tight dotted lines; and quadruple-locus variations = relaxed dotted lines. Note that all haplotypes structured as three clusters, which were defined as haplotype networks linking up to quadruple-locus variants. They appear as distinct colors.

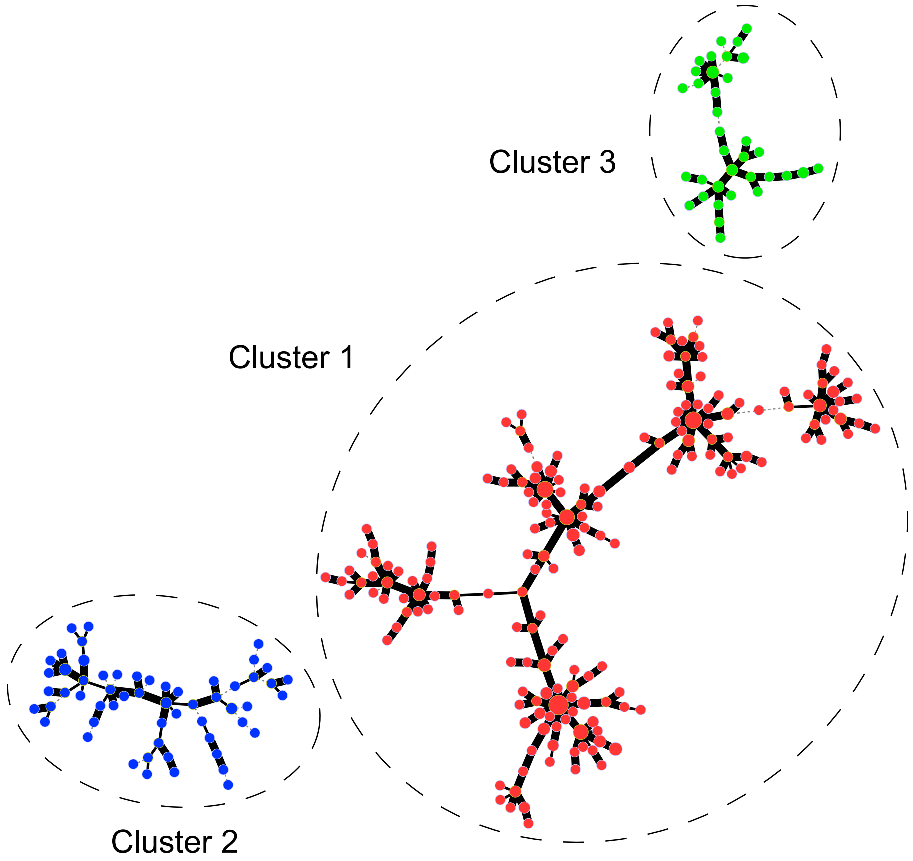

Supplement: Supplementary file 2 [file EVA-12-1523-s002.pdf]
