# Supplementary material for: Deciphering how plant pathogenic bacteria disperse and meet: Molecular epidemiology of Xanthomonas citri pv. citri at microgeographic scales in a tropical area of Asiatic citrus canker endemicity
Source: Evol Appl. 2019 Apr 10;12(8):1523–38. doi: 10.1111/eva.12788 (PMC6708428; doi:10.1111/eva.12788)

Fig. S3. Correlogram showing the spatial autocorrelation coefficient *r,* as a function of distance (for a distance class size of 4 m) and 95% confidence intervals about the null hypothesis of a random distribution of *X. citri* pv. *citri* in two Kaffir lime groves.

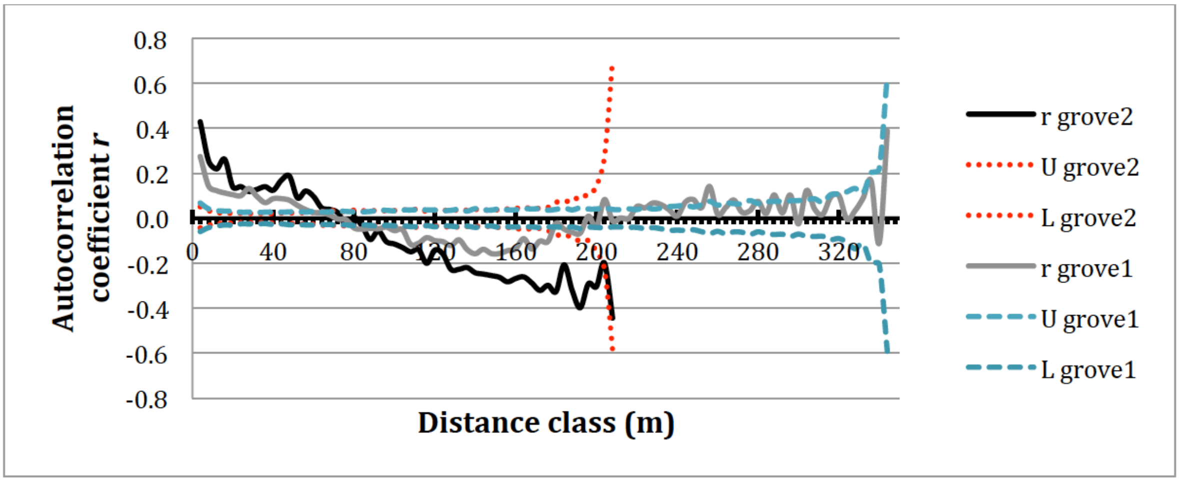

Supplement: Supplementary file 3 [file EVA-12-1523-s003.pdf]
