# Supplementary material for: Deciphering how plant pathogenic bacteria disperse and meet: Molecular epidemiology of Xanthomonas citri pv. citri at microgeographic scales in a tropical area of Asiatic citrus canker endemicity
Source: Evol Appl. 2019 Apr 10;12(8):1523–38. doi: 10.1111/eva.12788 (PMC6708428; doi:10.1111/eva.12788)

Fig. S4. Number of samples showing evidence of within-lesion polymorphism in canker lesions, using 14 microsatellite loci. Samples originated from two Kaffir lime groves.

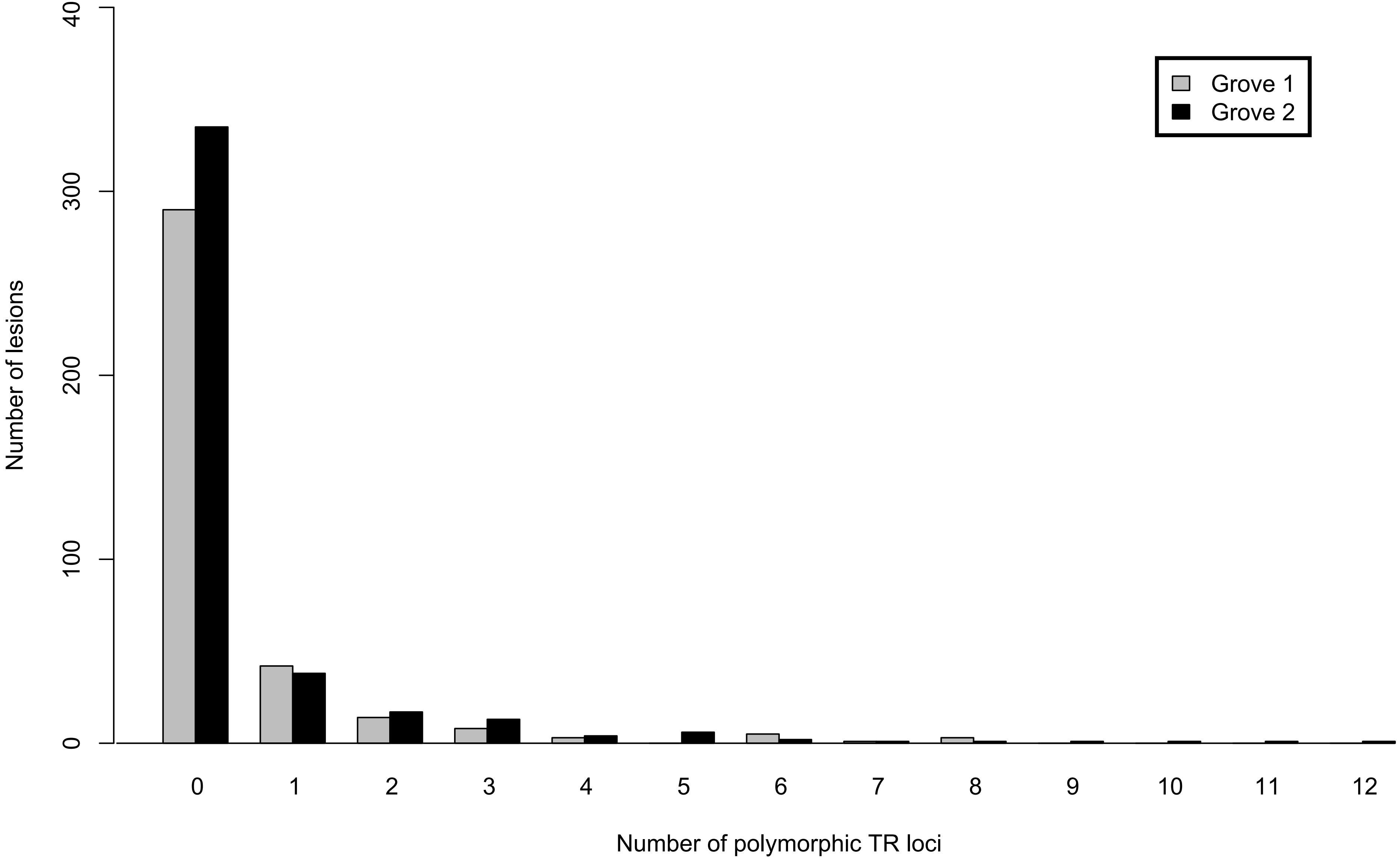

Supplement: Supplementary file 4 [file EVA-12-1523-s004.pdf]
