# Supplementary material for: Deciphering how plant pathogenic bacteria disperse and meet: Molecular epidemiology of Xanthomonas citri pv. citri at microgeographic scales in a tropical area of Asiatic citrus canker endemicity
Source: Evol Appl. 2019 Apr 10;12(8):1523–38. doi: 10.1111/eva.12788 (PMC6708428; doi:10.1111/eva.12788)
Supplement: Supplementary file 5 [file EVA-12-1523-s005.docx]

**Table S1.**Genetic differentiation among trees from citrus grove 2 estimated by R_ST_  (below the diagonal). Inter-trees distances (meters) are indicated above the diagonal.

|  | tree 1 | tree 2 | tree 3 | tree 4 | tree 5 | tree 6 |
| --- | --- | --- | --- | --- | --- | --- |
| tree 1 | 0 | 33.17 | 48.40 | 32.93 | 34.63 | 49.74 |
| tree 2 | 0.213*** | 0 | 15.04 | 13.04 | 30.41 | 27.5 |
| tree 3 | 0.054* | 0.358*** | 0 | 21.71 | 38.69 | 26.08 |
| tree 4 | 0.180*** | 0.120*** | 0.323*** | 0 | 17.80 | 17.92 |
| tree 5 | 0.315*** | 0.286*** | 0.368*** | 0.282*** | 0 | 21.71 |
| tree 6 | 0.482*** | 0.648*** | 0.498*** | 0.671*** | 0.087*** | 0 |

Significance level: * P ≤ 0.05 ; *** P ≤ 0.001

**Table S2.** Genetic differentiation estimated by R_ST_ among  branches (3 branches / tree; 6 trees) in citrus grove 2.

|  | Tree 1 | | | Tree 2 | | | Tree 3 | | | Tree 4 | | | Tree 5 | | | Tree 6 | | |
| --- | --- | --- | --- | --- | --- | --- | --- | --- | --- | --- | --- | --- | --- | --- | --- | --- | --- | --- |
| Branch | 1 | 2 | 3 | 4 | 5 | 6 | 7 | 8 | 9 | 10 | 11 | 12 | 13 | 14 | 15 | 16 | 17 | 18 |
| 1 | 0 |  |  |  |  |  |  |  |  |  |  |  |  |  |  |  |  |  |
| 2 | 0.098* | 0 |  |  |  |  |  |  |  |  |  |  |  |  |  |  |  |  |
| 3 | 0.010 NS | 0.208*** | 0 |  |  |  |  |  |  |  |  |  |  |  |  |  |  |  |
| 4 | 0.171*** | 0.415*** | 0.105*** | 0 |  |  |  |  |  |  |  |  |  |  |  |  |  |  |
| 5 | 0.215*** | 0.408*** | 0.173*** | 0.292*** | 0 |  |  |  |  |  |  |  |  |  |  |  |  |  |
| 6 | 0.146** | 0.375*** | 0.084* | 0.162*** | 0.055 NS | 0 |  |  |  |  |  |  |  |  |  |  |  |  |
| 7 | 0.057 NS | 0.141* | 0.066* | 0.232*** | 0.272*** | 0.220*** | 0 |  |  |  |  |  |  |  |  |  |  |  |
| 8 | 0.239*** | 0.029 NS | 0.348*** | 0.538*** | 0.525*** | 0.500*** | 0.231** | 0 |  |  |  |  |  |  |  |  |  |  |
| 9 | 0.083* | -0.016 NS | 0.197** | 0.408*** | 0.424*** | 0.382*** | 0.106* | 0.036 NS | 0 |  |  |  |  |  |  |  |  |  |
| 10 | 0.135** | 0.372*** | 0.103** | 0.178*** | 0.471*** | 0.381*** | 0.191*** | 0.511*** | 0.361*** | 0 |  |  |  |  |  |  |  |  |
| 11 | 0.137** | 0.375*** | 0.080* | 0.102*** | 0.413*** | 0.304*** | 0.155** | 0.505*** | 0.359*** | 0.067* | 0 |  |  |  |  |  |  |  |
| 12 | 0.169*** | 0.390*** | 0.072* | 0.154*** | 0.134** | 0.024 NS | 0.196*** | 0.506*** | 0.392*** | 0.372*** | 0.271*** | 0 |  |  |  |  |  |  |
| 13 | 0.347*** | 0.480*** | 0.284*** | 0.399*** | 0.386*** | 0.376*** | 0.196*** | 0.553*** | 0.467*** | 0.471*** | 0.368*** | 0.303*** | 0 |  |  |  |  |  |
| 14 | 0.194*** | 0.341*** | 0.137** | 0.218*** | 0.162** | 0.152** | 0.095* | 0.423*** | 0.327*** | 0.263*** | 0.198*** | 0.110* | 0.018 NS | 0 |  |  |  |  |
| 15 | 0.384*** | 0.513*** | 0.333*** | 0.472*** | 0.461*** | 0.448*** | 0.233*** | 0.580*** | 0.499*** | 0.570*** | 0.455*** | 0.382*** | -0.019 NS | 0.041 NS | 0 |  |  |  |
| 16 | 0.502*** | 0.595*** | 0.479*** | 0.743*** | 0.717*** | 0.710*** | 0.336*** | 0.645*** | 0.581*** | 0.863*** | 0.764*** | 0.664*** | 0.110*** | 0.164*** | 0.098*** | 0 |  |  |
| 17 | 0.489*** | 0.584*** | 0.467*** | 0.744*** | 0.707*** | 0.699*** | 0.319*** | 0.634*** | 0.570*** | 0.868*** | 0.769*** | 0.652*** | 0.093*** | 0.132* | 0.076*** | 0.057* | 0 |  |
| 18 | 0.453*** | 0.556*** | 0.415*** | 0.655*** | 0.629*** | 0.613*** | 0.271*** | 0.610*** | 0.541*** | 0.761*** | 0.656*** | 0.546*** | 0.041 NS | 0.080* | 0.052* | 0.150** | 0.080** | 0 |

NS not significant P > 0.05 ; * P ≤ 0.05 ; ** P ≤ 0.01 ; *** P ≤ 0.001

**Table S3.** Hierarchical AMOVA for *X. citri* pv. *citri* populations sampled from six trees in grove 2 (n = 552) and genotyped using MLVA-14 data based on size differences (R_ST_).

| **Source of variation** | **d.f.** | **Sum of squares** | **Variance components** | **Percentage of variation** | P-value |
| --- | --- | --- | --- | --- | --- |
| Among trees | 5 | 3728.180 | 7.2058 | 30.05 | <0.0001 |
| Among branches within trees | 12 | 919.900 | 2.00080 | 8.34 | <0.0001 |
| Within branches | 539 | 7962.161 | 14.77210 | 61.60 | <0.0001 |
| Total | 556 | 12610.241 | 23.97875 |  |  |
